# Probiotic Effects of *Lactobacillus fermentum* ZJUIDS06 and *Lactobacillus plantarum* ZY08 on Hypercholesteremic Golden Hamsters

**DOI:** 10.3389/fnut.2021.705763

**Published:** 2021-06-28

**Authors:** Dongting Yang, Wentao Lyu, Ziyi Hu, Jiting Gao, Zhiyao Zheng, Weijun Wang, Jenni Firrman, Daxi Ren

**Affiliations:** ^1^College of Animal Sciences, Institute of Dairy Science, Zhejiang University, Hangzhou, China; ^2^State Key Laboratory for Managing Biotic and Chemical Threats to the Quality and Safety of Agro-Products, Institute of Agro-Product Safety and Nutrition, Zhejiang Academy of Agricultural Sciences, Hangzhou, China; ^3^Zhejiang Yiming Food Co. Ltd., Wenzhou, China; ^4^Dairy and Functional Foods Research Unit, Eastern Regional Research Center, Agricultural Research Service, U.S. Department of Agriculture, Wyndmoor, PA, United States

**Keywords:** *in vivo*, probiotic potential, *Lactobacillus fermentum*, *Lactobacillus plantarum*, cholesterol-lowering effects, intestinal microbiota

## Abstract

Hypercholesteremia or high cholesterol is one of the important factors leading to atherosclerosis and other cardiovascular diseases. The application of probiotics with cholesterol-lowering characteristics has become increasingly popular over the past decade due to their contribution to human health. This study aimed to evaluate the probiotic effects of *Lactobacillus fermentum* ZJUIDS06 and *Lactobacillus plantarum* ZY08 on hyperlipidemic golden hamsters. A hyperlipidemic model was established through a high cholesterol diet in golden hamsters, after which lyophilized *Lactobacillus fermentum* ZJUIDS06 and *Lactobacillus plantarum* ZY08 were orally administered individually for 8 weeks. The physiological characteristics of golden hamsters and short chain fatty acid (SCFA) in the colon were assessed by automatic Biochemical Analyzer and gas choromatograph, respectively. A MiSeq sequencing-based analysis of the bacterial 16S rRNA gene (V3–V4 region) in the cecum content was performed to analyze the cecum microbiota. Correlations between sets of these variables were also investigated using the R package “corrplot.” Results showed that neither *Lactobacillus fermentum* ZJUIDS06 nor *Lactobacillus plantarum* ZY08 inhibited body weight increase. However, supplementation with *Lactobacillus fermentum* ZJUIDS06 for 8 weeks increased colon SCFA levels (*P* < 0.05), decreased serum low-density lipoprotein, total cholesterol, and triglycerides levels, and also induced changes in the cecum microbiota of hyperlipidemic golden hamsters. Remarkably, oral administration of *Lactobacillus fermentum* ZJUIDS06 increased the relative abundance of *Parabacteroides* in the cecum, which served as a biomarker for colon SCFA production and improvement of serum cholesterol levels. In a word, *Lactobacillus fermentum* ZJUIDS06 improved hyperlipidemia in golden hamsters, which correlated with an increase in SCFA levels and relative abundance of *Parabacteroides*, indicating its potential importance in functional foods that can help lower cholesterol.

## Introduction

Atherosclerosis is a type of cardiovascular disease that has become increasingly prevalent on a global scale and contributes to the etiology of multiple diseases, such as coronary heart disease, cerebral infarction, and peripheral vascular disease (1–3). The most commonly associated pathogenic factor for atherosclerosis is the increase in low-density lipoprotein cholesterol (LDL-C) levels in conjugation with a decrease in high-density lipoprotein cholesterol (HDL-C) levels (4, 5). The prescribed use of statin drugs reduces the risk of developing atherosclerotic cardiovascular disease (6, 7), cardiovascular-related death, and death in general, by lowering LDL-C levels (8–10). However, the use of statins on a regular basis has been associated with multiple adverse effects, including liver damage, liver necrosis, kidney damage, myopathy, and rhabodomyolysis (6, 11, 12), asks for the development of alternative agents or bioactives with cholesterol-lowering characteristics.

The administration of cholesterol-lowering probiotics has become increasingly popular over the past few decades due to their generally recognized as safe (GRAS) status and their contribution to the healthy microbiota of human mucosal surfaces. To date, a number of cholesterol-lowering strains have been isolated from feces of healthy people, fermented dairy products, and pickles, including *Lactobacillus plantarum, Lactobacillus fermentum, Lactobacillus acidophilus, Lactobacillus casei, Lactobacillus reuteri, Lactobacillus rhamnosus, Bifidobacterium*, and *Enterococcus faecium*. The LAB strains previously reported to exhibit the cholesterol-lowering effect *in vivo* mainly belong to *Lactobacillus* and *Enterococcus* (13, 14).

The cholesterol-lowering effects of *Lactobacillus fermentum* or *Lactobacillus plantarum* have been reported from both animal models and human clinical trials. *Lactobacillus fermentum* MJM60397 reduced the levels of serum triglycerides (TG) and LDL-C, and improved gene expression of LDL-R in livers of male ICR mice after a 7-week intervention period (15). Consumption of buffalo milk fermented by *Lactobacillus fermentum* improved serum lipids and biochemical indexes of livers in male Wistar rats (16). *Lactobacillus plantarum* EM fermented juice reduced the levels of serum TG, total cholesterol (TC), and LDL-C of Sprague-Dawley rats and improved the expression of 7 α-hydroxylase and LDL receptors in the rat liver (17). In addition, *Lactobacillus plantarum* colonizing the colon of rats reduced serum alanine aminotransferase (ALT), aspartate aminotransferase (AST), TC, TG, LDL, very low-density lipoprotein (VLDL), and the Atherogenic Index under hypercholesterolemic conditions (18). In human clinical trials, ingestion of *Lactobacillus fermentum* ME-3 for 4 weeks decreased serum TG and oxidized-LDL and increased serum HDL-C, and thus reducing the risk of developing cardiovascular disease and diabetes (19). Heat-inactivated *Lactobacillus plantarum* L-137 reduced the levels of serum TC, LDL-C, AST, and ALT in overweight people (20). In a human clinical trial, treatment with live *Lactobacillus plantarum* Q180 for 12 weeks decreased postprandial maximum concentrations of TG, LDL-C, Apo B-100, and Apo B-48 levels (21).

Several mechanisms for cholesterol reduction by lactic acid bacteria (LAB) have been proposed, such as deconjugation of bile salts by bile-salt hydrolase (BSH) (22–24), binding and incorporation of cholesterol to the LAB cellular surface (25–27), production of short-chain fatty acids (SCFAs) during the LAB growth (28, 29), and co-precipitation of cholesterol with deconjugated bile salts (30, 31). Nevertheless, the mechanism for cholesterol reduction by LAB needs to be studied on a case-to-case basis and the cholesterol-lowering effects of LAB still need to be elucidated.

In our previous study, two cholesterol-lowering probiotics, *Lactobacillus plantarum* ZY08 and *Lactobacillus fermentum* ZJUIDS06, were isolated from baby feces. Both strains demonstrated cholesterol-lowering effects *in vitro* (Supplementary Figure 1), were resistant to acid and bile salt, and had no antibiotic resistance. However, the *in vivo* cholesterol-lowering effects of these two strains were still unknown, and their effects on the intestine microbial community remained unclear. Therefore, the objectives of this study were to assess the effects of *Lactobacillus plantarum* ZY08 and *Lactobacillus fermentum* ZJUIDS06 on serum lipids, SCFA profiles, and gut microbiota in hyperlipidemic golden hamsters, and thus to provide deeper insights into the counter- hyperlipidemic effects of certain probiotics.

## Materials and Methods

### Bacterial Strains, Culture Conditions, and Gavage Administration

*Lactobacillus plantarum* ZY08 and *Lactobacillus fermentum* ZJUIDS06 (Supplementary Figure 2) were isolated from breastfed baby (6 months old) feces in Hangzhou, Zhejiang Province, China. The two strains were grown in MRS broth (Beijing Land Bridge Technology Co. Ltd., Beijing, China) and incubated anaerobically at 37°C for 18 h. *In vitro* cholesterol-lowering characteristics for the two strains (Supplementary Figure 1) were determined following previously described methods (32). The two strains were lyophilized (WECARE-BIO Biotechnology Co. Ltd., Jiangsu, China) and stored at −20°C until use.

### Golden Hamster Experimental

The *in vivo* experiment was conducted following a previous study with some modifications (33, 34). In this experiment, cholesterol (0.1%) and lard (10%) were added to the standard diet (Supplementary Table 1, Pluteng Biological Technology Co. Ltd., Shanghai, China) to produce the high cholesterol diet. Golden hamsters (*Mesocricetus auratus*, Vital River Laboratory Animal Technology Co. Ltd., Beijing, China) were fed the high cholesterol diet to develop the mixed hyperlipidemia model. Animal care and experimental procedures were approved prior to initiation (#17426), and the guidelines set by the Animal Care and Use Committee of Zhejiang University was followed.

**Figure 1:**
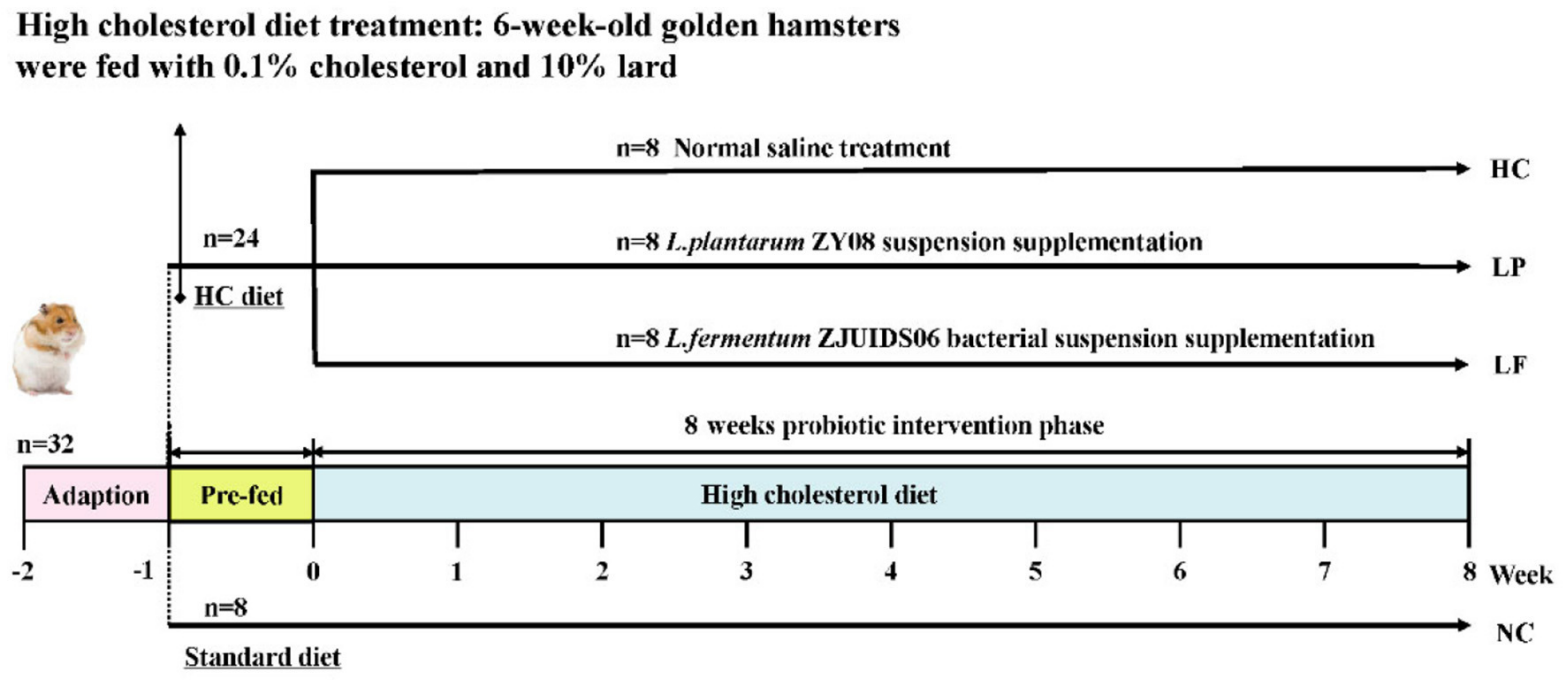
Experimental design of animal study.

Golden hamsters were selected as the animal model for this study because hamsters synthesize and excrete cholesterol and bile acids in a manner similar to that of humans, and they have become a standard model for evaluating the cholesterol-decreasing efficacy of probiotic strains (34–36). Male hamsters are considered a better model than females for developing hyperlipidemia and evaluating the cholesterol- decreasing efficacy because they are more susceptible to a high-fat diet induced weight gain (37). Accordingly, male hamsters were chosen as a model to assess the cholesterol-lowering effect of LAB *in vivo*. A flow chart summarizing the aims of our study can be found in Figure 1. A total of 32 male golden hamsters, 6 weeks old, were fed a standard diet for 1 week to allow them to adapt to their new environment. Subsequently, 24 hamsters were fed the high cholesterol diet for 8 weeks, and the other eight hamsters were maintained using a standard diet for 8 weeks and were considered as the negative control group (NC group). To initiate the experimental phase, the 24 hamsters fed the high cholesterol diet were randomly assigned to the following three groups according to their body weight: High cholesterol positive control group (HC group), *Lactobacillus plantarum* ZY08 intervention group (LP group), and *Lactobacillus fermentum* ZJUIDS06 intervention group (LF group). During the experimental period, golden hamsters in the NC group and HC group were given 1 mL normal saline per 100 g body weight per day. Hamsters in the LP group were given 1 mL of *Lactobacillus plantarum* ZY08 suspension (10^9^ CFU/mL) per 100 g body weight per day, while hamsters in the LF group were given 1 mL *Lactobacillus fermentum* ZJUIDS06 suspension (10^9^ CFU/mL) per 100 g body weight per day. The viable counts of lyophilized bacteria were enumerated on MRS agar by surface plating on a weekly basis. Lyophilized bacteria were suspended in saline and the viable cell counts in the suspension used for gavage administration were ~10^9^ CFU/mL (35, 38, 39).

Body weight and food intake were measured every week and blood samples were collected every 2 weeks. After an 8-week intervention period, all golden hamsters were euthanized, and dissected before blood, liver, kidney, and intestine tract samples were collected and frozen −80°C until analyzed.

### Serum Lipids Determination

Blood samples were collected every 2 weeks from the intraorbital venous plexus of the golden hamsters using a flat-ending capillary (0.5 mm diameter) tube. The blood was stored at 4°C overnight and centrifuged at 3000× g for 10 min to harvest the serum for lipid profiling. The amounts of TC, LDL-C HDL-C, and TG in the serum were determined by automatic Biochemical Analyzer (#3100, Hitachi, Ltd., Tokyo, Japan), according to the manufacturer's procedure.

### Histological Examination of the Liver

The livers of the golden hamsters were frozen in liquid nitrogen immediately after dissection. The tissue was covered by an OCT embedding agent (Tissue-Tek O.C.T. Compound 4583, SAKURA Finetek USA, Torrance, USA), frozen, and then cut out using Cryotome E (Thermo Fisher Scientific, Waltham, USA). A coverslip (Servicebio Technology Co. Ltd., Wuhan, China) was applied to attach the tissue. Finally, the hematoxylin-eosin staining was performed, and the section was observed using an oil immersion lens (Eclipse E100, Nikon, Corp., Tokyo, Japan) and scanned by Pannoramic MIDI (3DHISTECH^™^ Ltd., Budapest, Hungary).

### Determination of SCFA

SCFA in the colon content were determined following a previously described method with some modification (40). After dissection, the segmented colon sections were squeezed with sterile forceps, and the contents were removed and stored in cryopreservation tubes at −80°C. The colon contents were diluted five-fold with ultrapure water and vortexed for 3 min. Next, the suspension was rested for 5 min and then centrifuged at 4°C, 5000× g for 20 min. One milliliter of supernatant was mixed with 20 μL chromatogram grade phosphoric acid (Shanghai Aladdin Biochemical Technology Co. Ltd.), and the mixture was injected into a chromatographic vial (WondaVial, Shimadzu, Corp., Kyoto, Japan) through a 0.45 μm membrane filter for gas chromatography.

The gas chromatography machine (Shimadzu, Corp., Kyoto, Japan) consisted of an AOC-20S auto sampler and a GC-2010 equipped with a flame ionization detector. A SH-stabliwax (#227-36305-2, 30×0.25×0.25, Shimadzu, Corp., Kyoto, Japan) highly polar column was installed on the GC with nitrogen as the carrier gas at a flow rate of 3 mL/min. The sample injection volume was 0.2 μL with a split injection ratio of 50 and an injection temperature of 200°C. The ethyl acetate was injected as a blank solvent between every sample to remove any memory effects. The initial column temperature was set at 80°C and held for 1 min, then increased to 170°C at a rate of 8°C/min, then immediately increased to 220°C at a rate of 20°C/min and maintained for 4 min. The total time was 18.75 min. Finally, the content of SCFAs was calculated according to the SCFA standard curve, which was calibrated by the external standard method.

### Tissue Weight

At the end of the experiment, the adipose tissue surrounding liver, kidney, and epididymis were collected, washed with PBS, and dried with clean filter paper. The tissues were then weighed by using an electronic balance (BSA124, Sartorius, Inc., Gottingen, Germany) and recorded.

### DNA Extraction and Cecum Microbiota Analysis

The cecum content usually contains the highest absolute number and diversity of microorganisms in the gastrointestinal tract (41), and is thus particularly useful for the analysis of microbiota. After dissection, the segmented cecum was squeezed with sterile forceps to remove the content, which was stored in cryopreservation tubes at −80°C. DNA from the cecum content was extracted using the Fast DNA SPIN extraction kit (MP Biomedicals, Inc., Santa Ana, USA) following the manufacturer's protocol. Quantity and quality of the extracted DNA were measured using the NanoDrop DN-1000 spectrophotometer (Thermo Fisher Scientific, Inc., Waltham, USA) and agarose gel electrophoresis, respectively. PCR amplification of the bacterial 16S rRNA genes V3–V4 region was performed using the forward primer 338F (5′-ACTCCTACGGGAGGCAGCA-3′) and the reverse primer 806R (5′-GGACTACHVGGGTWTCTAAT-3′) (Personal Biotechnology Co. Ltd., Shanghai, China) (42–44). Sample-specific 7-bp barcodes were added to the primers for multiplex sequencing. The PCR components consisted of 5 μL of Q5 reaction buffer (5×), 5 μL of Q5 High-Fidelity GC buffer (5×), 0.25 μL of Q5 High-Fidelity DNA polymerase (5 U/μL), 2 μL (2.5 mmol/L) of dNTPs, 1 μL (10 μmol/L) of each forward and reverse primer, 2 μL of DNA template, and 8.75 μL of ddH_2_O. Thermal cycling covered initial denaturation at 98°C for 2 min, followed by 25 cycles including denaturation at 98°C for 15 s, annealing at 50°C for 30 s, and extension at 72°C for 30 s, with a final extension of 5 min at 72°C. The Agencourt AMPure Beads (Beckman Coulter, Inc., Indianapolis, USA) were applied for PCR amplicon purification and a PicoGreen dsDNA Assay Kit (Invitrogen, Thermo Fisher Scientific, Waltham, USA) was used for quantification. Based on quantification, amplicons were gathered in equal amounts, and pair-end 2 × 300 bp sequencing was performed using the Illlumina MiSeq platform with the MiSeq Reagent Kit v3 at Shanghai Personal Biotechnology Co. Ltd. (Shanghai, China). The sequencing data were uploaded to the Sequence Read Archive (SRA) of NCBI and can be viewed with the following accession code: PRJNA727412.

Bioinformatics were applied to the sequencing data using QIIME2 (45) with slight modifications. Briefly, raw sequence data were demultiplexed using the demux plugin and then the primer removed with the cut adapt plugin (46). Sequences were quality filtered, de-noised, merged, and chimeras were removed using the DADA2 plugin (47). Mafft (48) was applied to align the non-singleton amplicon sequence variants (ASVs) and then the results were applied to construct a phylogeny with fasttree2 (49). The diversity plugin was applied to estimate the Alpha-diversity metrics Chao1 (50). The observed species, Shannon (51, 52), and Simpson (53), and the beta diversity metrics, weighted UniFrac (54), unweighted UniFrac (55), and Bray-Curtis dissimilarity were identified. All samples were rarefied to 56,522 sequences. The classify-sklearn naïve Bayes Taxonomy classifier in feature-classifier plugin (45) was applied to assign Taxonomy to ASVs set against the SILVA Release 132 Database (56).

### Statistical Analysis

Experiments were performed in biological triplicates. All data are expressed as means ± SD. Differences between variables were tested for significance by one-way ANOVA with a Tukey's Test (General parameter data) or a Kruskal-Wallis test (Non-parametric data) using IBM SPSS version 24.0 (International Business Machines, Corp., Armonk, USA). Differences at *P* < 0.05 were considered significant. QIIME2 and R packages (v3.6.3) were used to analyze the sequencing data. The ASV table in QIIME2 was used to calculate the ASV-level alpha diversity indices, such as Chao1 richness estimator, Observed species, Shannon diversity index, Simpson index, Faith's PD, Pielou's evenness, Good's coverage, and to visualize box plots. The richness and evenness of ASVs between the samples were compared by the generated ASV-level ranked abundance curves. Bray-Curtis metrics (57), non-metric multidimensional scaling (NMDS), and unweighted pair-group method with arithmetic means (UPGMA) hierarchical clustering (58) were applied to investigate the structural variation of microbial communities across samples in Beta diversity analysis. PERMANOVA (Permutational multivariate analysis of variance) (59) and ANOSIM (Analysis of similarities) (60, 61) in QIIME2 were applied to assess the significance of differentiation for the microbiota structure among groups. LEfSe (Linear discriminant analysis effect size) was applied to detect differentially abundant taxa around the groups in the default parameters (62). The correlation between the genus level abundance of cecum microbiota and SCFA was analyzed by Spearman's correlation coefficient and plotted using the R package “corrplot.”

## Results

### Golden Hamster Body Weights and Daily Dietary Intake

**Figure 2:**
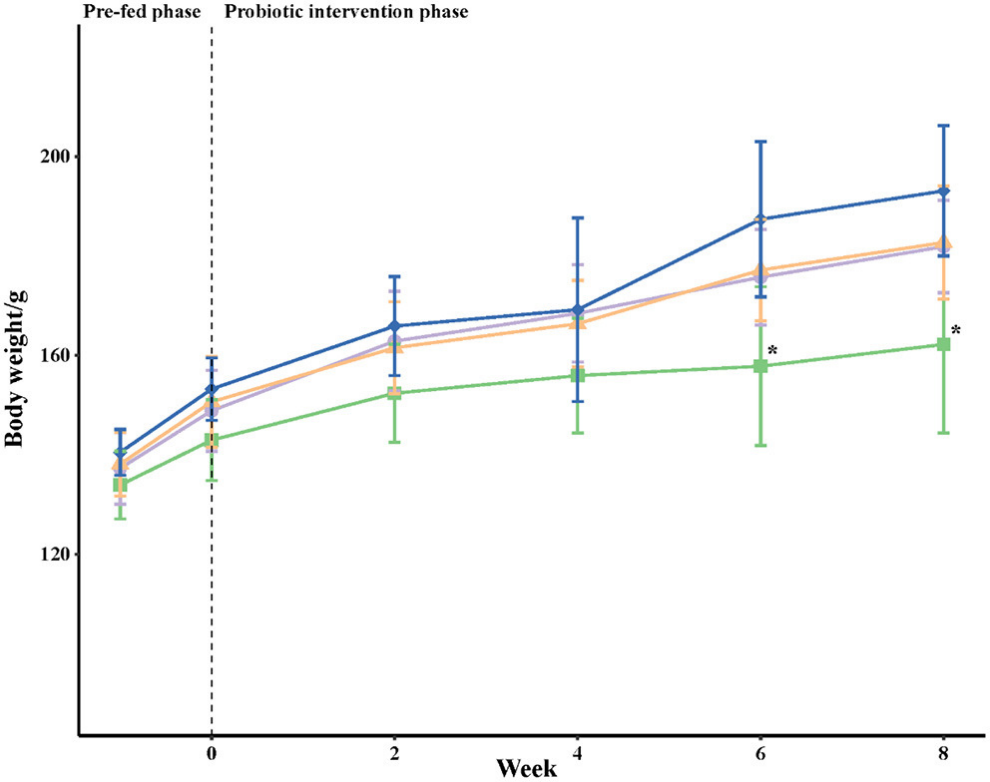
Body weights (g) of golden hamsters over time. The body weight for each golden hamster was measured once a week; each point represents the mean per group ± SD (*n* = 8). Statistical analysis was performed using one-way ANOVA and a Tukey's test. At each time point, an asterisk (^*^) symbol indicates that the mean value was significantly lower than the mean value of the HC group. NC, negative control group; HC, positive control group; LP, *Lactobacillus plantarum* ZY08 supplemented group; LF, *Lactobacillus fermentum* ZJUIDS06 supplemented group. NC, HC, LP, and LF are colored in green (■), purple (•), orange (▴), and blue (♦), respectively.

The body weight for each golden hamster was recorded weekly and the means for each group were calculated (Figure 2). In the pre-fed and early intervention periods, the mean values of body weight were comparable among all groups. However, the mean body weights for golden hamsters in the positive control group (HC), *Lactobacillus plantarum* supplemented group (LP), and *Lactobacillus fermentum* supplemented group (LF) were higher than those in the negative control group (NC) after 6 and 8 weeks of the experiment. Similar results for body weights were observed in the three groups fed the high cholesterol diet (HC, LP, and LF). There was no significant difference in the daily nutritional intake between all groups.

### Effect of LAB on Serum Lipids

**Figure 3:**
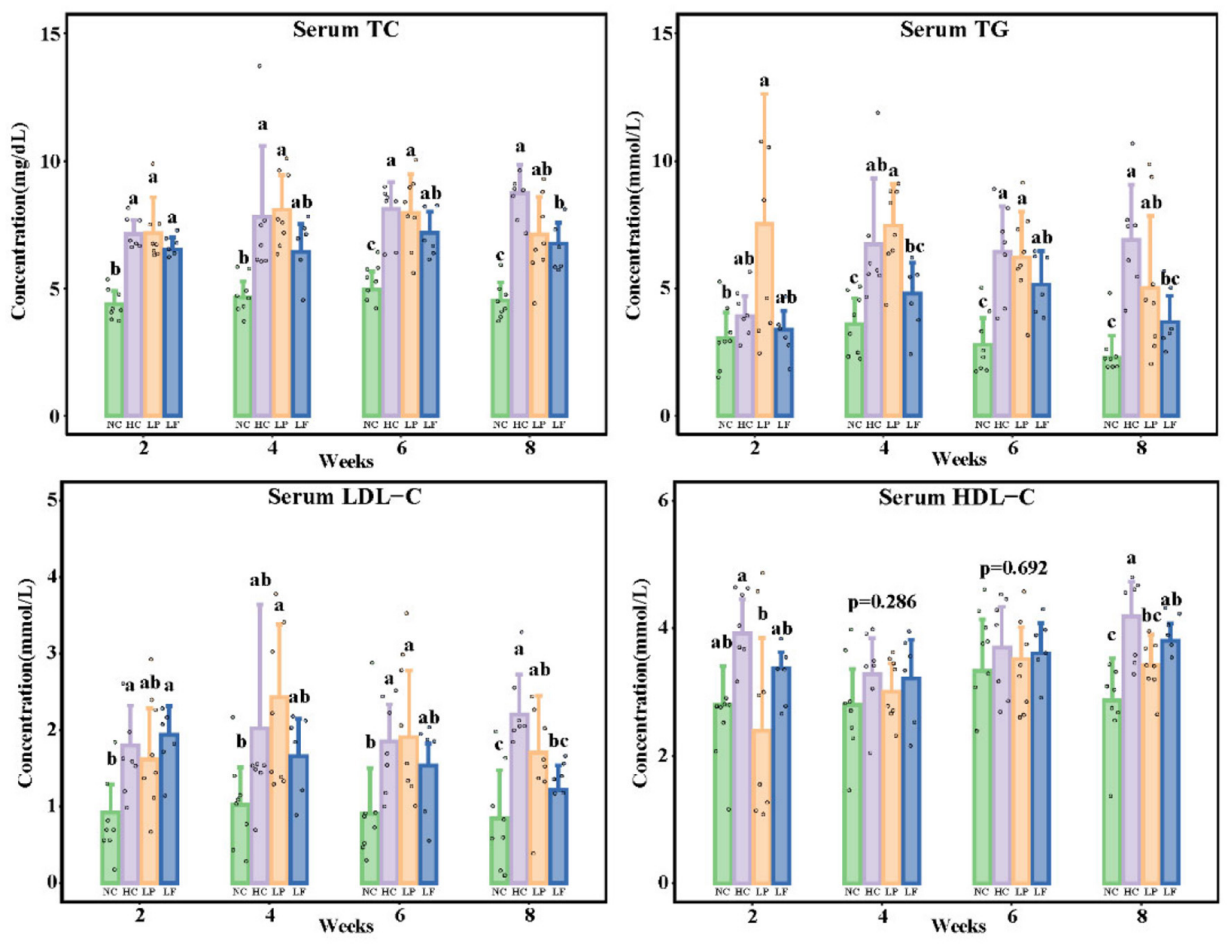
Serum lipid levels of golden hamsters. Groups annotated with *a, b, c* are significantly different with *P* < 0.05 as determined by one-way ANOVA and a Tukey's test. TC, total cholesterol; TG, triglyceride; LDL-C, low-density lipoprotein cholesterol; HDL-C, high-density lipoprotein cholesterol; NC, negative control group; HC, positive control group; LP, *Lactobacillus plantarum* ZY08 supplemented group; LF, *Lactobacillus fermentum* ZJUIDS06 supplemented group. NC, HC, LP, and LF are colored in green, purple, orange, and blue, respectively.

Serum lipids, including TC, LDL-C, HDL-C, and TG were determined every 2 weeks. Exposing the golden hamsters to a high cholesterol diet for 2 weeks increased serum TC and LDL-C levels, indicating that the addition of 0.1% cholesterol and 10% lard to the feed was suitable for inducing hypercholesteremia. After 8 weeks, the serum TC, TG, HDL-C, and LDL-C levels were overall significantly different among all the groups (Figure 3). Ingestion of *Lactobacillus fermentum* ZJUIDS06 for 8 weeks reduced serum TC and TG levels in the golden hamsters fed the high cholesterol diet by 1.97 mg/dL and 3.21 mmol/L, respectively, and reduced serum LDL-C by 44.8%, while *Lactobacillus plantarum* ZY08 did not have such an effect. Ingestion of *Lactobacillus fermentation* ZJUIDS06 did not affect the serum HDL-C levels in golden hamsters fed the high cholesterol diet, while ingestion of *Lactobacillus plantarum* ZY08 reduced the HDL-C levels.

### Liver Histology

**Figure 4:**
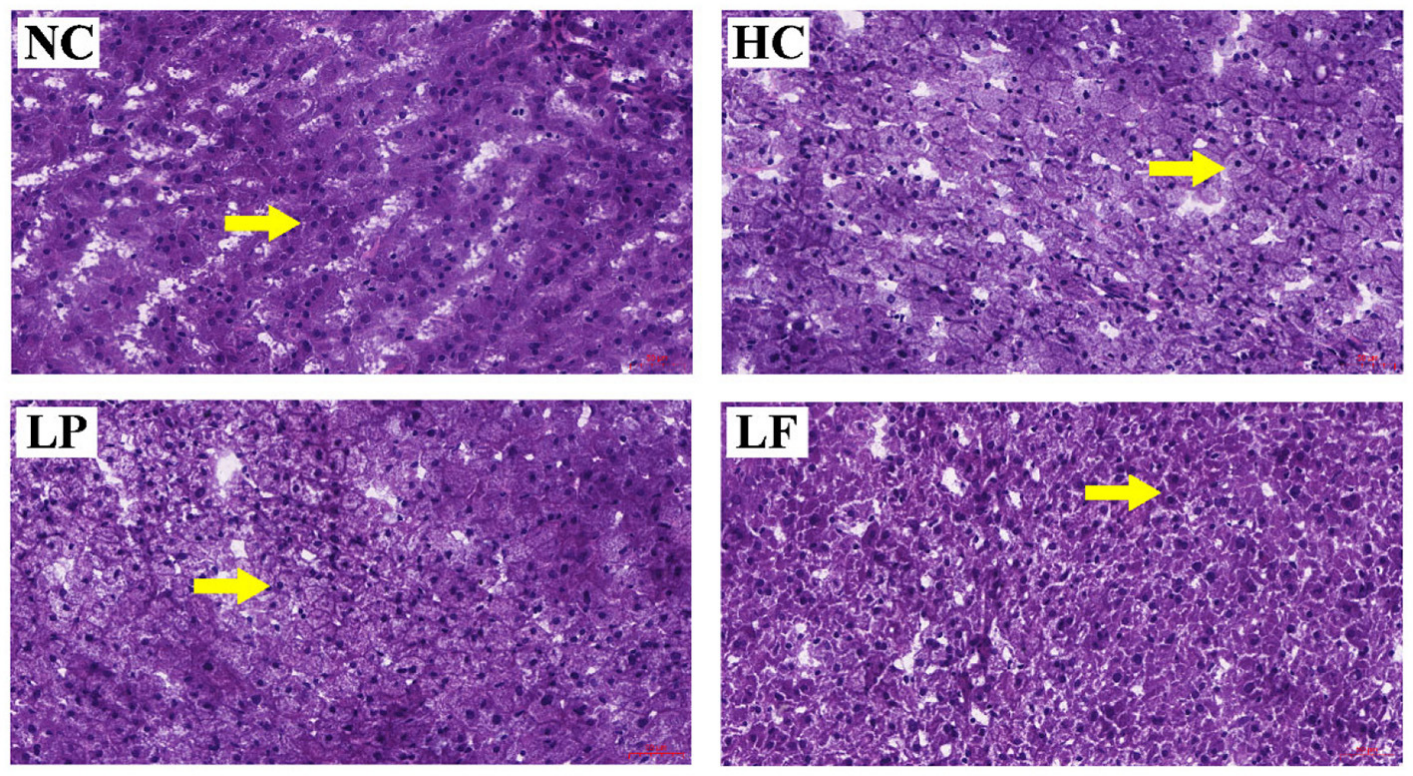
Histological staining of liver tissues from hyperlipidemic golden hamsters after 8-weeks of treatment. Arrows indicate the situation of cytoplasm near the nucleus. Specimens were visualized and image captured using light microscopy (H & E stain, magnification: ×200, Scale bar, 50 μm). NC, negative control group; HC, positive control group; LP, *Lactobacillus plantarum* ZY08 supplemented group; LF, *Lactobacillus fermentum* ZJUIDS06 supplemented group.

Histopathological analysis was performed on the harvested livers using hematoxylin-eosin staining to assess the effects of LAB supplementation on hepatocyte steatosis (Figure 4). The cytoplasm near the nucleus was compact in the hepatocytes of golden hamsters in the NC group. In the HC and LP group, a large number of lipid droplets appeared in the cytoplasm near the nucleus of the hepatocytes. Remarkably, ingestion of *Lactobacillus fermentum* ZJUIDS06 reduced the number of lipid droplets that were present in the cytoplasm near the nucleus of the hepatocytes.

### Effect of LAB on SCFA

**Figure 5:**
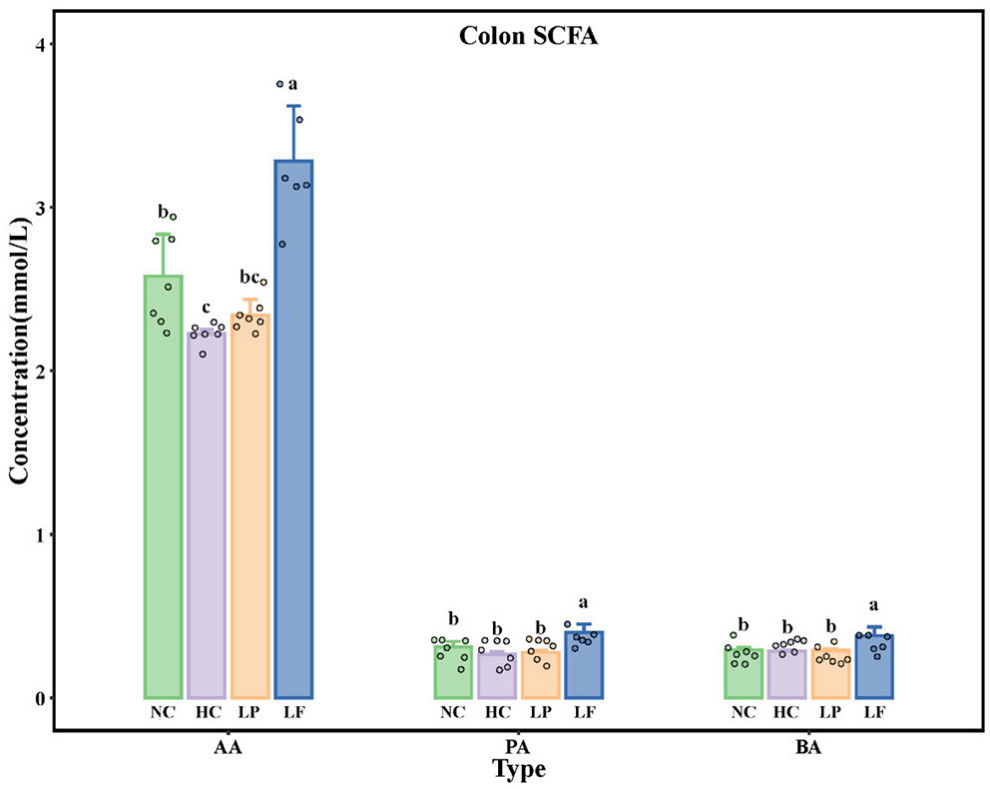
Colon SCFA levels in golden hamsters after 8 weeks of treatment. Groups annotated with *a, b, c* differed significantly with *P* < 0.05 as determined by one-way ANOVA and a Tukey's test. AA, acetic acid; PA, propionic acid; BA, butyric acid; NC, negative control group; HC, positive control group; LP, *Lactobacillus plantarum* ZY08 supplemented group; LF, *Lactobacillus fermentum* ZJUIDS06 supplemented group. NC, HC, LP, and LF are colored in green, purple, orange, and blue, respectively.

The concentrations of acetic acid, butyric acid, and propionic acid in colon contents of golden hamsters are presented in Figure 5. The amount of total SCFAs in the NC group was lower than that of the HC group. Golden hamsters supplemented with *Lactobacillus fermentum* ZJUIDS06 had higher levels of total SCFAs compared to any other group. Ingestion of *Lactobacillus fermentum* ZJUIDS06 administration for 8 weeks increased acetic acid, propionic acid, and butyric acid by 1.06, 0.13, and 0.10 mmol/L, respectively, while ingestion of *Lactobacillus plantarum* ZY08 did not have similar effects.

### Effect of LAB on Tissue Weight

Liver and the epididymal fat pad (EFP) are the two major adipose tissue depots in golden hamsters (63). Therefore, the liver and epididymal fat pad (EFP) weights were recorded to assess the effects of LAB on fat accumulation in the hamsters (Supplementary Table 2). The weights of these organs were comparable among the HC, LP, and LF groups. The liver and EFP weight of golden hamsters in the HC, LP, and LF groups were higher than those in the NC group.

### Effect of LAB on Cecum Microbiota

To assess the effects of *Lactobacillus fermentum* ZJUIDS06 and *Lactobacillus plantarum* ZY08 intervention on the cecum microbiota of hyperlipidemic golden hamsters (Supplementary Figure 3), a MiSeq sequencing-based analysis of bacterial 16S rRNA (V3–V4 region) in cecum content was performed. After being spliced and optimized, 27 samples were delineated into 40,453 OTUs at a 95% similarity level with distance-based OTUs and richness, and rarefaction and Shannon index analysis indicating that the sequencing depth covered rare new phylotypes and most of the diversity (Supplementary Figure 4).

**Figure 6:**
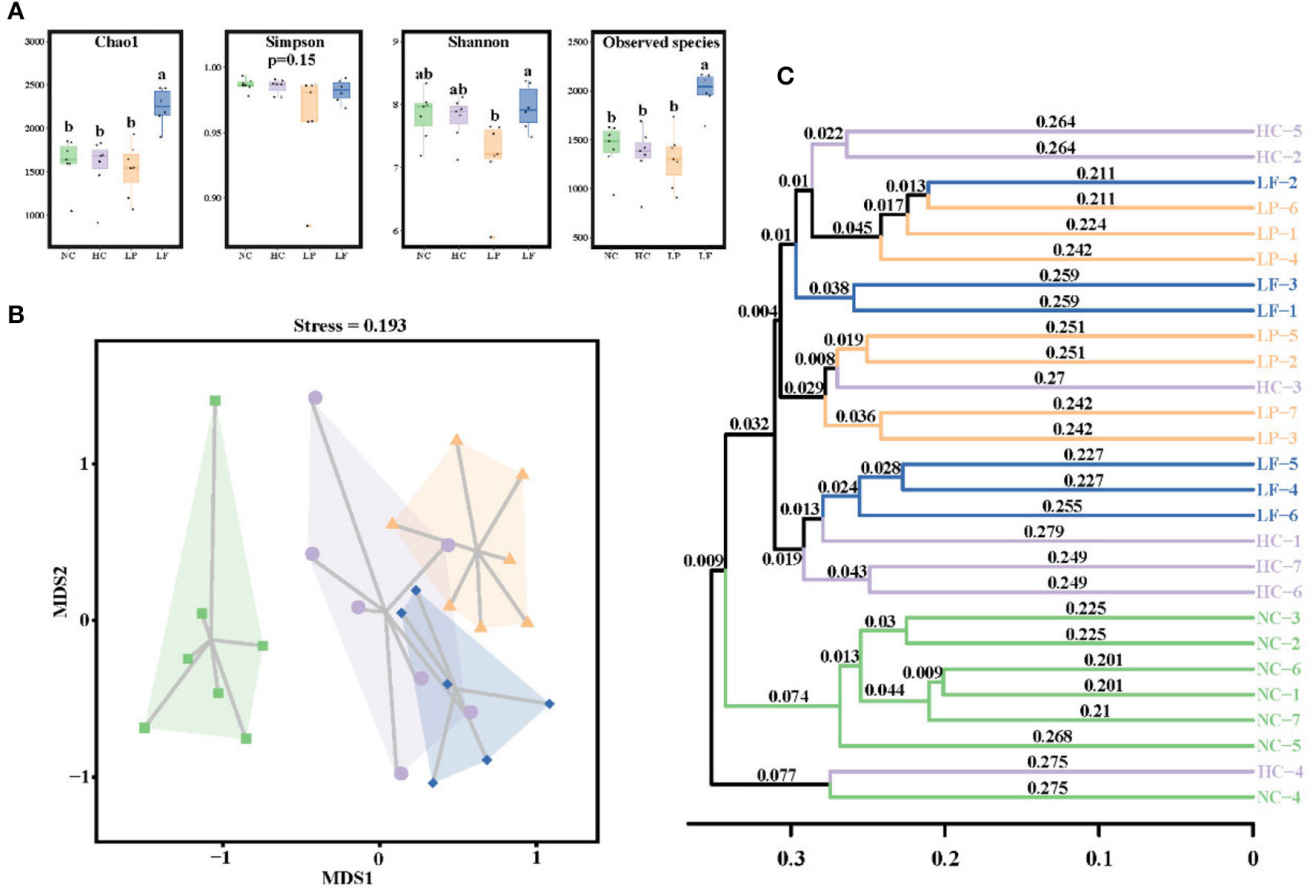
**(A)** Box plot depicting alpha diversity in the experimental groups of golden hamsters. Groups annotated with *a, b* significantly differed with *P* < 0.05, respectively, as determined by a Kruskal-Wallis and Dunn's test. **(B)** NMDS based on Bray-Curtis distance. **(C)** Clustering tree depicting the samples clustering according to their similarity. NC, negative control group; HC, positive control group; LP, *Lactobacillus plantarum* ZY08 supplemented group; LF, *Lactobacillus fermentum* ZJUIDS06 supplemented group. NC, HC, LP, and LF are colored in green (■), purple (•), orange (▴), and blue (♦), respectively.

The caecal microbiota community was first assessed by analyzing species richness, or the number of species in a community, and species diversity, which is the number of species and abundance of each species that live in a particular location (64) (Figure 6A). The observed species and Chao indices of the LF group were significantly higher than the other groups, indicating that the *L. fermentum* ZJUIDS06 intervention increased cecal microbiota richness. However, the Simpson and Shannon indices showed no differences between any of the two groups, indicating that the LAB intervention did not influence the diversity of cecal microbiota.

Next, a Multidimensional Scaling (NMDS) analysis was applied to visualize the differences in community structure between the groups. NMDS is similar to a Principal coordinates analysis (PCoA) analysis, but MNDS analysis is decomposed by dimensionality reduction, and the data structure is simplified, so that the distribution characteristics of the samples can be described using a specific distance scale (65). Here, NMDS analysis revealed a distinct clustering of microbiota composition between the standard diet group (NC) and the high cholesterol diet groups (HC, LP, LF) (Figure 6B). The significant separation between groups was confirmed using an unweighted pair-group method with arithmetic means (UPGMA) hierarchical clustering (Figure 6C) which showed the similarity between samples in the form of a hierarchical tree and the clustering effect by branch length. An analysis of similarities (ANOSIM) revealed that the overall microbiota structure differed significantly between groups (Supplementary Table 3), indicating that the two LAB interventions induced different shifts in the structure of the caecal microbiota community.

**Figure 7:**
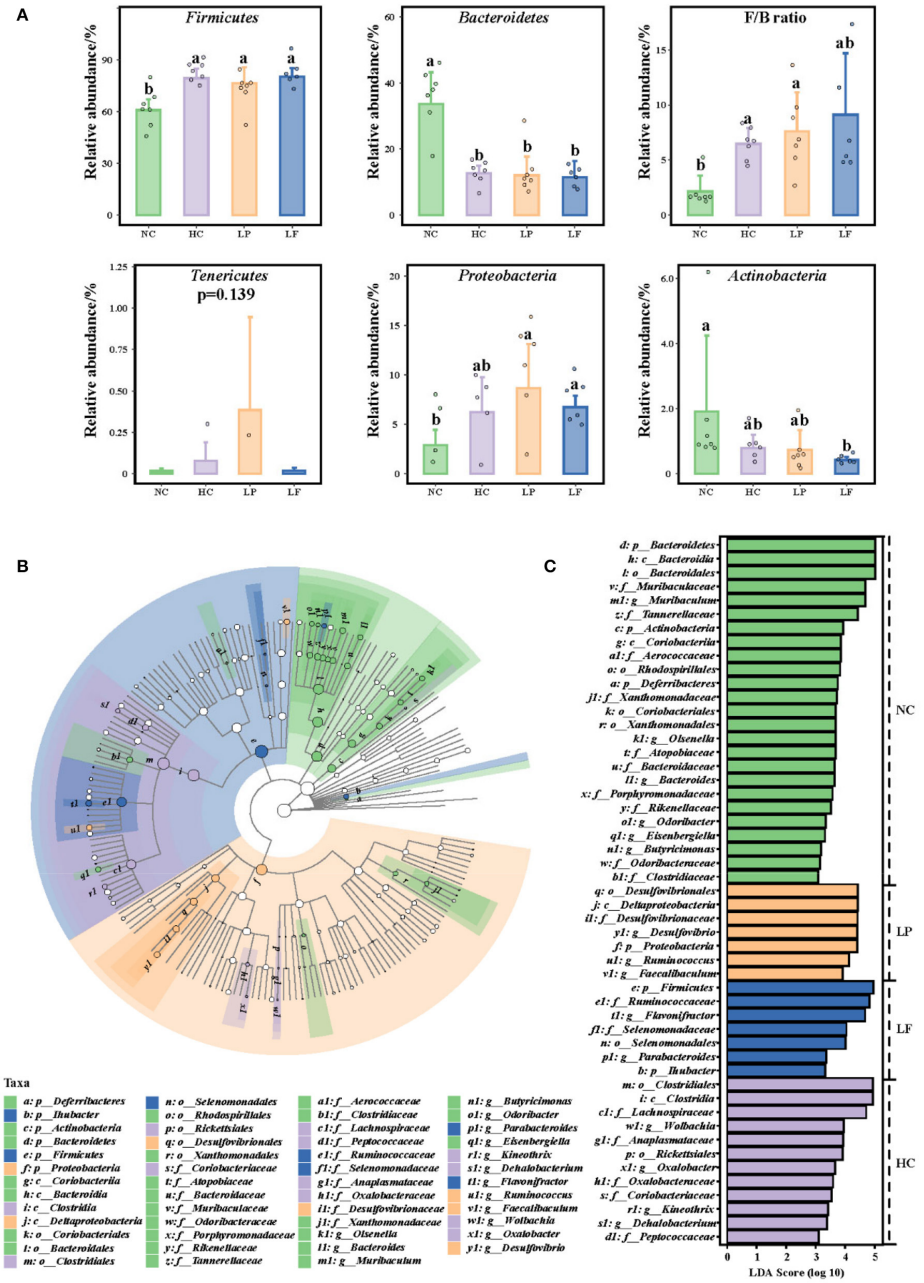
**(A)** Caecum microbial composition of golden hamsters at the phylum level. **(B)** Taxa Lefse cladogram. **(C)** LDA Score (LDA >2). Groups annotated with letters *a, b* were significantly different with *p* < 0.05 as determined by a Kruskal-Wallis test and FDR correction. NC, negative control group; HC, positive control group; LP, *Lactobacillus plantarum* ZY08 supplemented group; LF, *Lactobacillus fermentum* ZJUIDS06 supplemented group. NC, HC, LP, and LF are colored in green, purple, orange, and blue, respectively.

Finally, discriminant taxonomic markers were identified with linear discriminant analysis effect size (LEfSe) using the non-parametric factorial Kruskal–Wallis *H*-test (Figure 7). The LEfSe analysis resulted in three parts: The abundance histogram showed the specific distribution of significantly enriched species in different groups of the samples (Figure 7A); The species classification cladogram showed the taxonomic hierarchical distribution of significantly enriched species from the phylum to the genus level for each group of the samples (Figure 7B); the distribution histogram displayed the LDA value (LDA >2) of significantly different species, which is used to identify the significantly enriched taxa for each group and the important species identified (Figure 7C). Based on the results of the LEfSe analysis, *Firmicutes* and *Bacteroidetes* were the dominant phyla in the cecum of all groups. The cecum microbiota of golden hamsters fed a high cholesterol diet was characterized by an increased *Firmicutes*-to-*Bacteroidetes* ratio (Figure 7A). Remarkably, at the genus level, *Lactobacillus fermentum* ZJUIDS06 administration increased the relative abundance of *Parabacteroides, Flavonifractor*, and *Lactobacillus plantarum* ZY08 increased that of *Faecalibaculum, Ruminococcus*, and *Desulfovibrio*.

### Correlation Analysis

**Figure 8:**
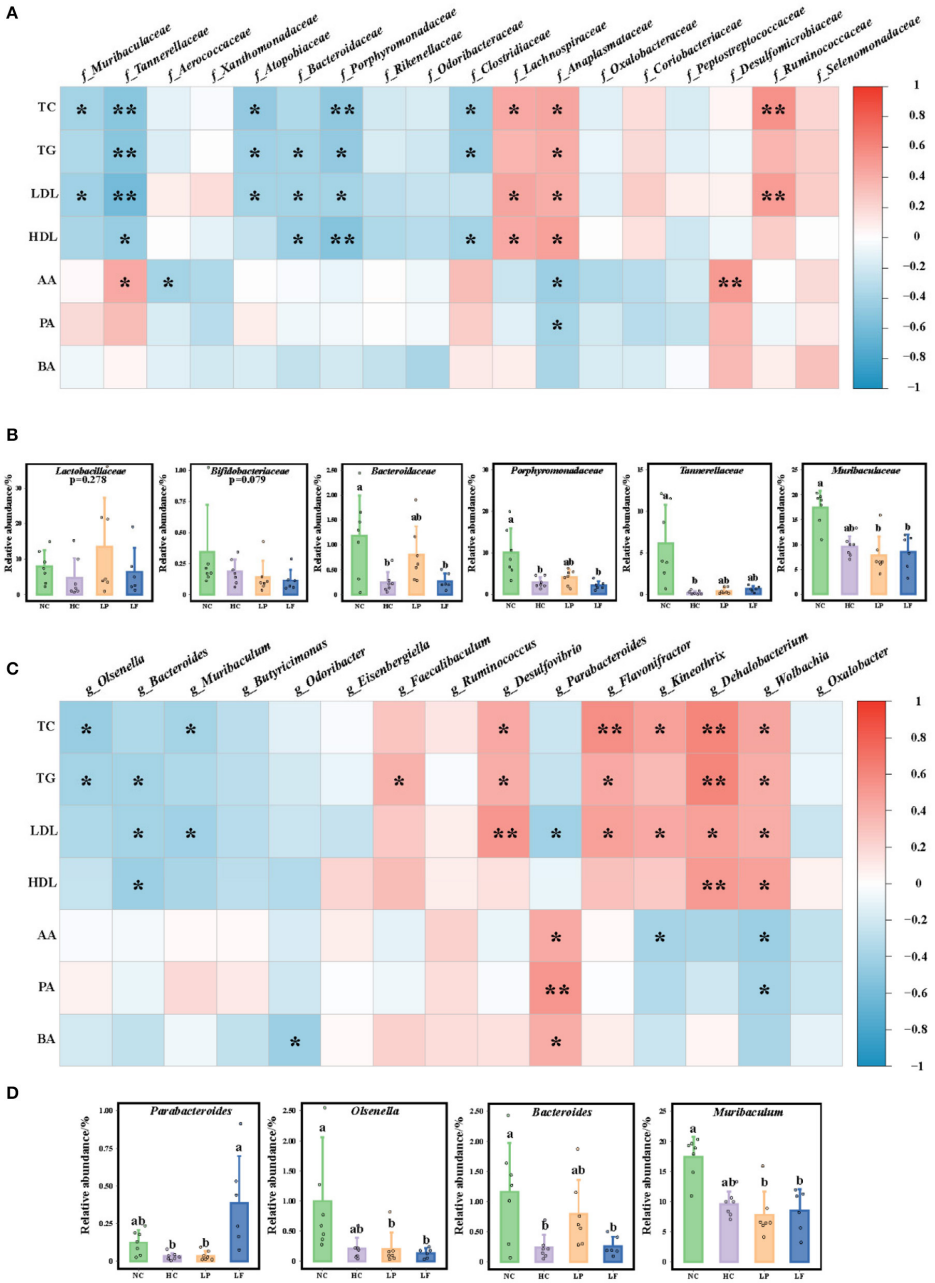
**(A)** Heat map of Spearman's correlation coefficients between serum lipid indexes, colon SCFA, and marker taxa at the family level (f_bacteria). **(B)** The relative abundance of taxa at the family level that had a significantly negative correlation with the serum lipid indexes including *Lactobacillaceae* and *Bifidobacteriaceae*. **(C)** Heat map of Spearman's correlation coefficient between serum lipid indexes, colon SCFA, and marker taxa at the genus level (g_bacteria). **(D)** The relative abundance of taxa at the genus level that had a significantly negative correlation with serum lipid indexes. Symbols ^*^ and ^**^ represent *P* < 0.05 and <0.01, respectively. NC, negative control group; HC, positive control group; LP, *Lactobacillus plantarum* ZY08 supplemented group; LF, *Lactobacillus fermentum* ZJUIDS06 supplemented group; NC, HC, LP, and LF are colored in green, purple, orange, and blue, respectively. TC, serum total cholesterol; TG, serum triglyceride; LDL, serum LDL-C; HDL, serum HDL-C; AA, acetic acid in colon content; PA, propionic acid in colon content; BA, butyrate acid in colon content.

To demonstrate whether the identified biomarkers were correlated with serum lipid indexes or cecum SCFAs, we performed an association analysis using the R package “corrplot” (Figure 8). At the family level, six of 10 marker taxa in the NC group, *Muribaculaceae, Tannerellaceae, Atopobiaceae, Bacteroidaceae, Porphyromonadaceae*, and *Clostridiaceae*, showed negative correlations with serum lipid indexes (Figure 8A). Three of five marker taxa in the HC group, *Lachnospiraceae, Anaplasmataceae*, and *Ruminococcaceae*, showed positive correlations with serum lipid indexes. Only *Desulfomicrobiaceae* in the LP group showed a positive correlation with acetic acid with a Spearman's correlation coefficient of 0.504. The classes significantly correlated with serum lipid reduction and *Lactobacillaceae* and *Bifidobacteriaceae* are shown in Figure 8B.

At the genus level, three of the six identified biomarker taxa from the NC group, *Olsenella, Bacteroides*, and *Muribaculum*, showed negative correlations with serum lipid indexes. *Kineothrix, Dehalobacterium*, and *Wolbachia* from the HC group, *Flavonifractor* from the LF group, and *Desulfovibrio* from the LP group showed significantly positive correlations with serum lipid indexes. Interestingly, only *Parabacteroides* was enriched in the LF group presenting a negative correlation with LDL-C, as well as a positive correlation with SCFA levels, with a Spearman's correlation coefficient of 0.430 for acetic acid, 0.534 for pentanoic acid, and 0.416 for butyric acid, respectively (Figure 8C). In addition, *Lactobacillus fermentum* ZJUIDS06 administration increased both the relative abundance of *Parabacteroides* (Figure 8D) and the SCFA concentration in the colon content (Figure 8A).

## Discussion

Ingestion of LAB did not affect body weights of hyperlipidemic golden hamsters, while daily supplementation with *Lactobacillus fermentum* ZJUIDS06, at the dosages of 10^9^ CFU per 100 g body weight for 8 weeks, significantly reduced LDL-C, TC, and TG levels in the hyperlipidemic golden hamsters. The lack of a decreasing body weight effect following LAB administration in golden hamsters may be related to the feeding methods used. In previous studies where golden hamsters were exposed to unpredictable chronic mild stress or LAB administration, ingestion of probiotics significantly decreased the body weight gain (66–68). However, in the studies where feeding was unrestricted but monitored, probiotic intervention had no effect on body weight gain (38, 39, 66, 69), which was consistent with our findings here. Remarkably, *Lactobacillus fermentum* ZJUIDS06 exhibited cholesterol-lowering effect while *Lactobacillus plantarum* ZY08 did not have such effect. The variation in cholesterol-lowering effect between these two strains may relate to their different interactions with the host, and still needs to be further elucidated (70). The observation that the reduction of LDL-C, TC, and TG levels by *Lactobacillus fermentum* ZJUIDS06 in hyperlipidemic golden hamsters positively correlated with the levels of colon SCFAs, indicated that oral administration of *Lactobacillus fermentum* ZJUIDS06 may reduce serum lipids by inducing increased colon SCFAs. The effects of SCFAs on cholesterol metabolism in cellular models, hyperlipidemic animal models, and in human clinical trials have been well-documented (citation). Previously, butyrate was found to increase the activity of the liver X receptor ABCG5 and G8 expression and to decrease NPC1L1 expression in Caco-2 cells (71). Concentration changes of SCFA indirectly activated ApoA-I expression with PPARα transactivation, increased transcription of PPARα and CPT1 and decreased transcription of KEAP1 in HepG2 cells (29). The dietary supply with SCFAs decreased serum lipids and promoted fecal excretion of bile acids in hyperlipidemic hamsters through up-regulation of SREBP2, LDLR, and CYP7A1 expression in the liver (28) and reduced the serum lipids in freshly weaned pigs by up-regulating the expression of hepatic FAS, CPT-1α, and SREBP-1 (72). Accordingly, the increase in colon SCFAs observed in this study may relate to the decrease in the serum lipids in hyperlipidemic golden hamsters. However, the role of SCFAs in mediating serum lipids still needs to be elucidated.

In previous reports, the cholesterol-lowering intervention strategies used in hyperlipidemic rodents increased the relative abundance of *Lactobacillus* or *Bifidobacteria* and decreased the ratio of *Firmicutes* to *Bacteroides* (39, 73–75). However, interventions used in this study did not have such effects (Figure 8B). The discrepancy between our research and other reports may relate to variations in the intestinal segments or animal models (41). Remarkably, in this research, *Parabacteroides* was the key symbiotic genus negatively correlated with LDL-C and positively correlated with colon SCFAs. Species of *Parabacteroides* have been reported as symbiotic bacteria that can alleviate obesity and metabolic dysfunction in mice (76). *Parabacteroides goldsteinii* relates to the anti-obesity effects of polysaccharides isolated from *Hirsutella sinensis* and water extract of *Ganoderma lucidum mycelium* in high-fat-diet (HFD) fed mice (77, 78). Oral treatment of HFD fed mice with live *P. goldsteinii* reduced obesity and was associated with increased adipose tissue thermogenesis. *P. goldsteinii* is a novel probiotic bacterium that may be used to treat obesity and associated metabolic disorders (78). These findings provide evidence that ingestion of *Lactobacillus fermentum* ZJUIDS06 may reduce serum lipids by enriching the commensal bacteria *Parabacteroides*.

Our results show that the oral administration of *Lactobacillus fermentum* ZJUIDS06 was not only positively correlated with *Parabacteroides*, but also with increased levels of SCFAs. The results of previous studies have found that some strains of *Parabacteroides* can produce SCFAs. For example, *Parabacteroides acidifaciens* sp. nov. ferments glucose into acetate acid, propionate, isobutyrate, and isopentanoate *in vitro* (79). However, the effects of *Parabacteroides* on *in vivo* SCFAs production remain inconclusive. Only one study found that oral administration of mice with alive *Parabacteroides distanonis* did not affect the level of acetate acid, propionate, isobutyrate, isopentanoate, and pentanoic acid in feces, but increased the level of jejunal succinic acid (76). Taken together, the correlation between *Parabacteroides* and *in vivo* SCFAs production still deserves further validation.

In conclusion, uptake of *Lactobacillus fermentum* ZJUIDS06 and *Lactobacillus plantarum* ZY08 did not prevent body weight gain in golden hamsters fed on a high cholesterol diet. However, oral administration of live *Lactobacillus fermentum* ZJUIDS06 in hyperlipidemic golden hamsters significantly increased colon SCFAs, and decreased the serum levels of LDL-C, TC, and TG, without affecting serum HDL-C, thus improving the colon SCFAs and serum lipid profiles. Both probiotics significantly altered the cecum microbiome, and the reduction of serum lipids following administration of *Lactobacillus fermentum* ZJUIDS06 was positively correlated with the relative abundance of *Parabacteroides*, which are commensal intestinal bacteria with probiotic characteristics. Our results give rise to a deeper understanding of the serum cholesterol-decreasing effects of certain probiotics.

## Data Availability Statement

The sequencing data was uploaded to the Sequence Read Archive (SRA) of NCBI and can be visited via accession number: PRJNA727412.

## Ethics Statement

The animal study was reviewed and approved by the Animal Care and Use Committee of Zhejiang University.

## Author Contributions

DY, ZH, WL, and DR: research design. DY and JG performed *in-vivo* experiments. DY, JG, and ZZ collected the sample and data. DY and ZH analyzed the data. ZH, JF, WW, WL, and DR revised the paper. All authors participated in the conception, design of the study, read, and approved the final manuscript.

## Conflict of Interest

WW was employed by Zhejiang YIMING food CO. LTD. The remaining authors declare that the research was conducted in the absence of any commercial or financial relationships that could have led to a potential conflict of interest.
