# Supplementary material for: Probiotic Effects of Lactobacillus fermentum ZJUIDS06 and Lactobacillus plantarum ZY08 on Hypercholesteremic Golden Hamsters
Source: Front Nutr. 2021 Jun 28;8:705763. doi: 10.3389/fnut.2021.705763 (PMC8273167; doi:10.3389/fnut.2021.705763)
Supplement: Supplementary file 1 [file Data_Sheet_1.pdf]

Support material

Table S1. Standard Diet Composition (g/kg)

Components	Standard diet
Moisture⩽	100
Crude protein⩾	205
Crude fat⩾	40
Crude fiber⩽	50
Crude ash⩽	80
Calcium	10-18
Total phosphorus	6-12
Calcium : Total phosphorus	1.2-1.7
Lysine⩾	13.2
Methionine + Cystine⩾	7.8

Complied with Chinese national standard GB14924.3-2010

Table S2. Organ weight after necropsy

Organ weight	NC	HC	LP	LF	SEM	p-value
Liver (g)	5.45[b]	8.89[a]	8.97[a]	8.36[a]	0.34	<0.01
EFP (g)	4.19[b]	6.31[a]	6.52[a]	5.96[a]	0.28	<0.01
Relative liver (%)	3.42[b]	4.88[a]	4.64[a]	4.57[a]	0.02	<0.01
Relative EFP (%)	2.60[b]	3.47[a]	3.35[ab]	3.25[ab]	0.12	0.034

a, b: P-value<0.05, EFP: Epididymal Fat Pad; Organ index was expressed as "relative organ /%": the ratio of the organ weight to the animal body weight

Table S3. Pairwise Anosim results

		Sample size	Permutations	R	p-value	q-value
**Group 1**	**Group 2**					
**HC**	**LF**	13	999	0.293651	0.011	0.011
	**LP**	14	999	0.272109	0.005	0.006
	**NC**	14	999	0.598639	0.001	0.003
**LF**	**LP**	13	999	0.506614	0.002	0.003
	**NC**	13	999	0.812169	0.002	0.003
**LP**	**NC**	14	999	0.823129	0.002	0.003
**HC**	**LF**	13	999	0.293651	0.011	0.011

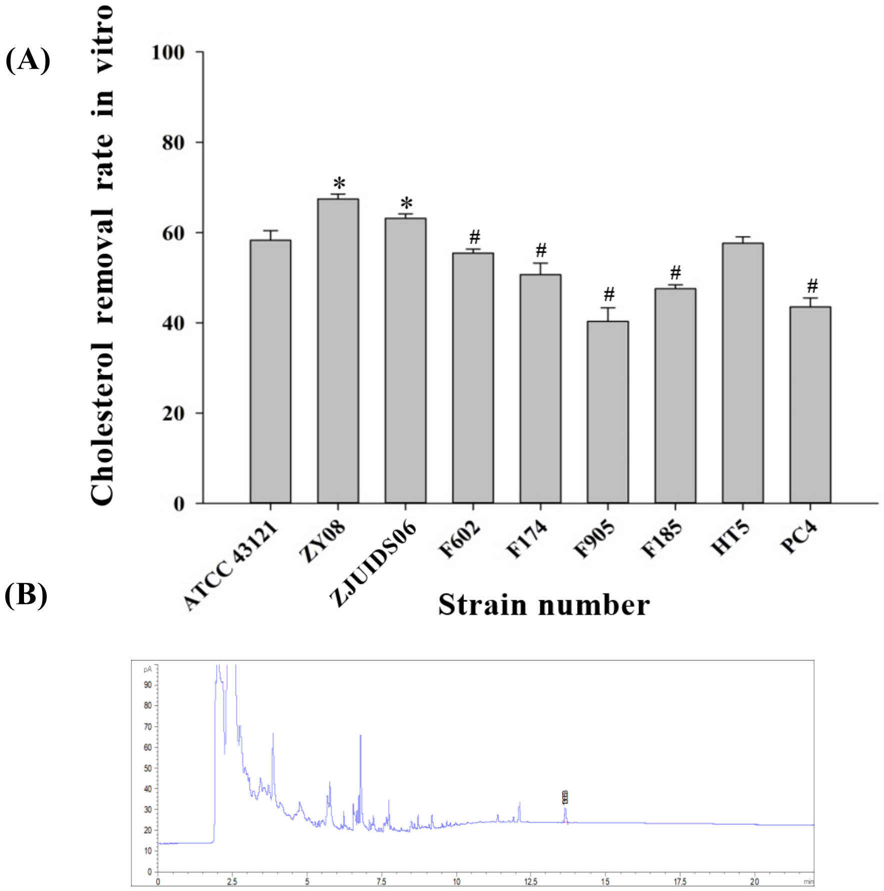

Figure S1. (A) Results of screening for cholesterol-lowering LAB strains; X-axis: Strain number; Y-axis: Cholesterol removal rate in vitro (%); (B) GC pattern of cholesterol; the strains with F and ZY08 and ZJUIDS06 are from infant feces, PC is from pickles, HT is from Jinhua ham. A * symbol indicates that the representative value was significantly higher than that of ATCC 43121 ($p < 0.05$); A# symbol indicates that the representative value was significantly lower than that of ATCC 43121 ($p < 0.05$).

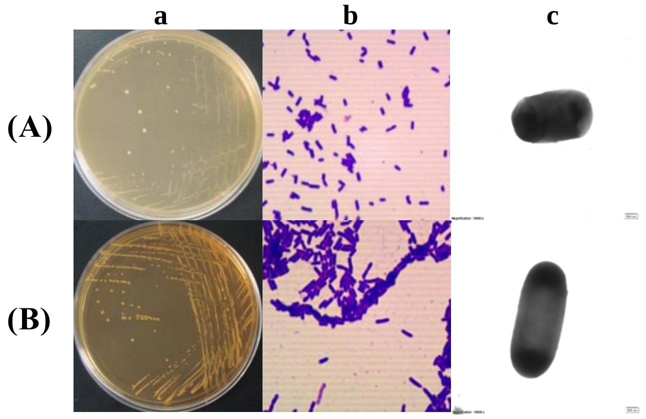

Figure S2. (a) Colony morphology; (b) Cell morphology (1000×); (c) Cell morphology (10000×)

(A) *Lactobacillus plantarum* ZY08; (B) *Lactobacillus fermentans* ZJUIDS06;

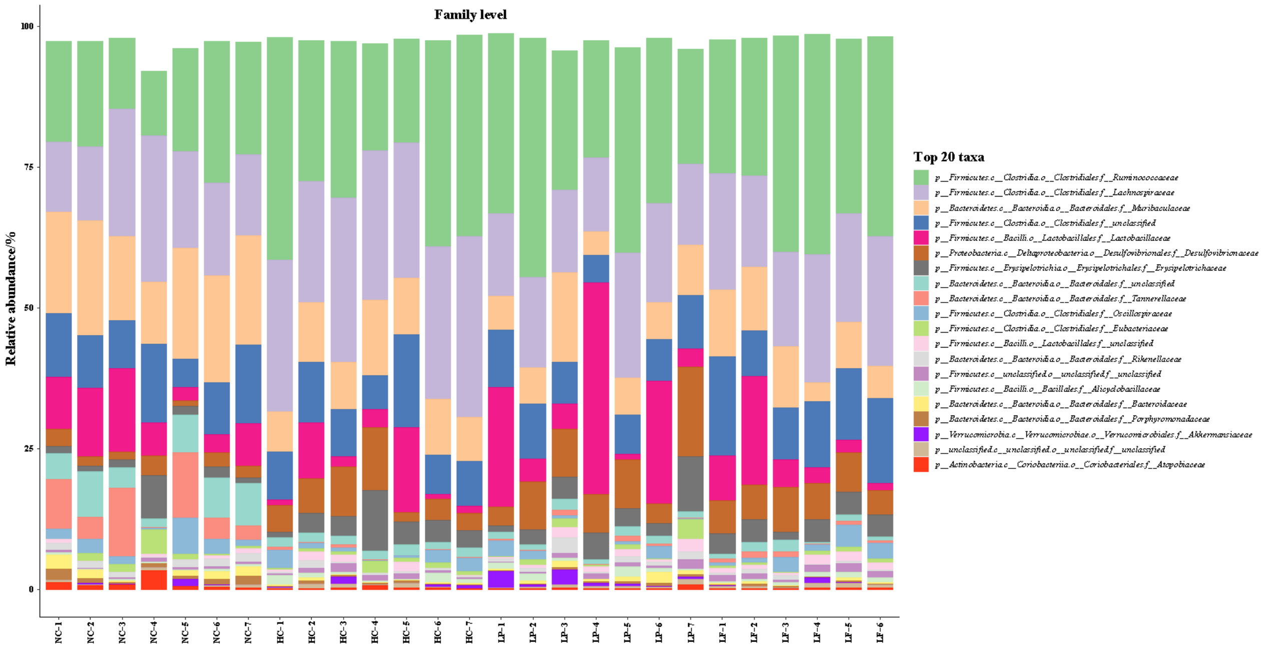

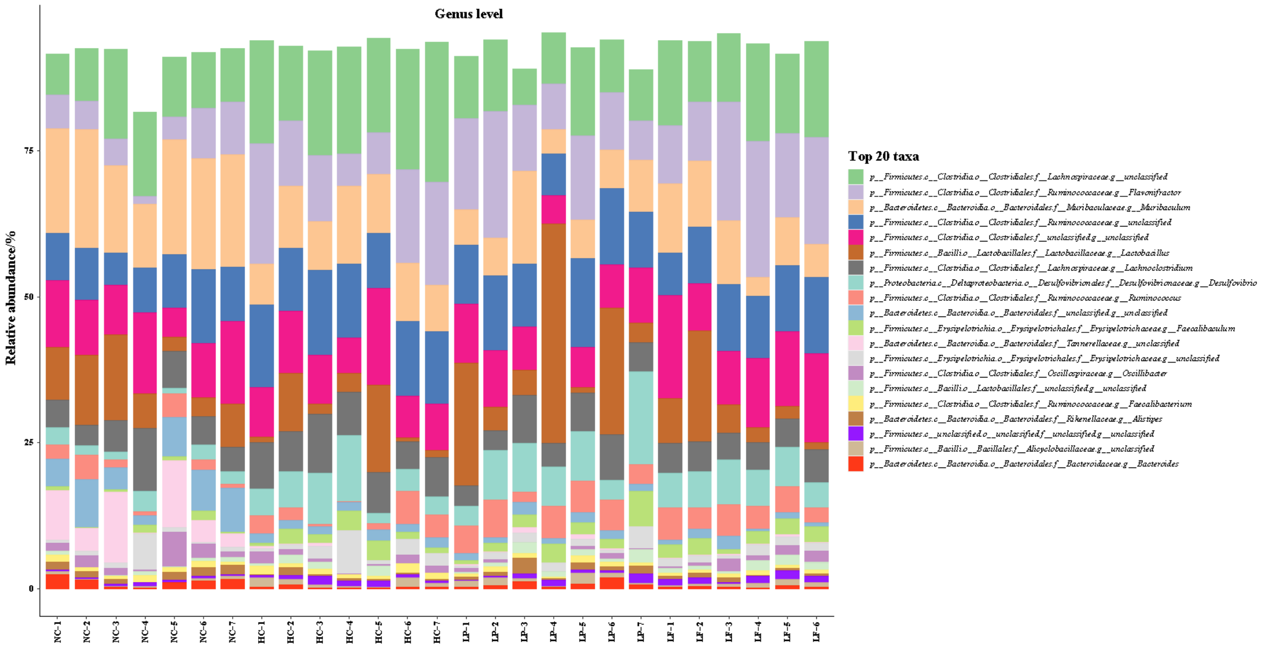

Figure S3. The top 20 taxa of cecum microbial composition at family and genus level. X-axis: different individuals of golden hamster.

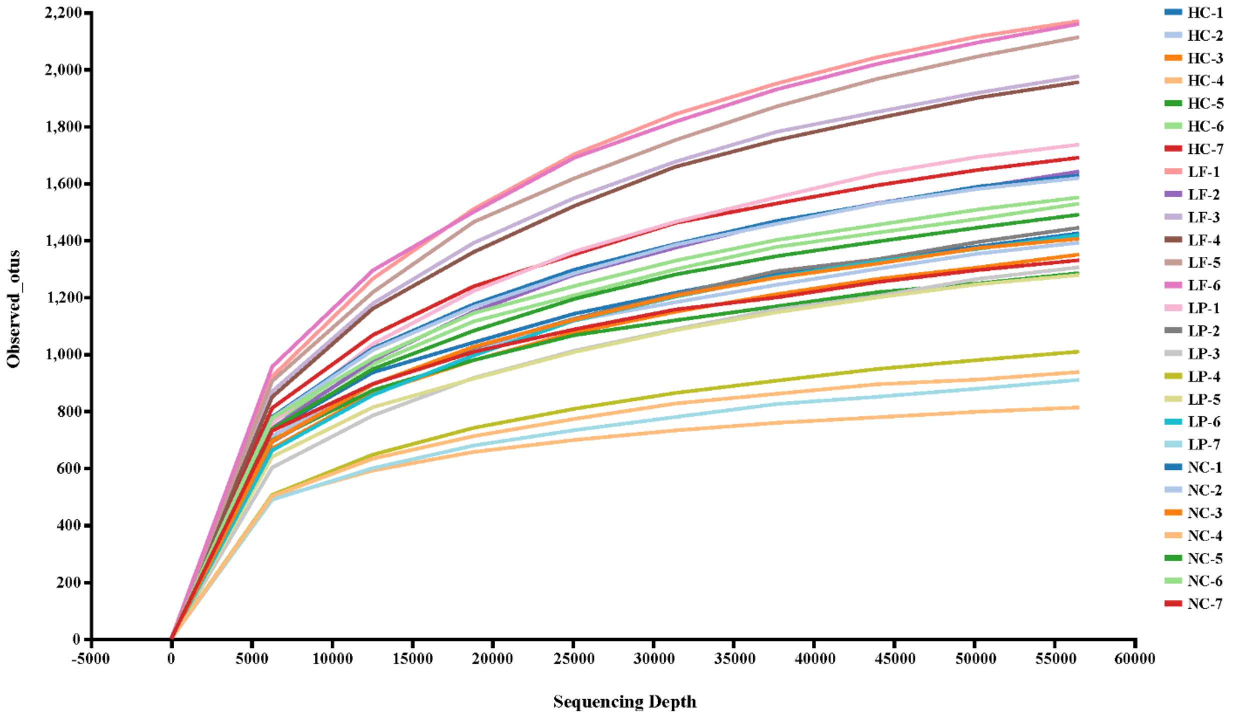

Figure S4. Alpha rarefaction curve.

Sequence Read Archive (SRA) was uploaded in NCBI #PRJNA727412:

https://www.ncbi.nlm.nih.gov/bioproject/PRJNA727412

16S rDNA sequence of *Lactobacillus plantarum* ZY08 (1509bp), provided by Zhiyao Zheng:

CTGGCTCAGGACGAACGCTGGCGGCGTGCCTAATACATGCAAGTCGAACGAACTCTGGTA

TTGATTGGTGCTTGCATCATGATTTACATTTGAGTGAGTGGCGAACTGGTGAGTAACACGT

GGGAAACCTGCCCAGAAGCGGGGGATAACACCTGGAAACAGATGCTAATACCGCATAAC

AACTTGGACCGCATGGTCCGAGTTTGAAAGATGGCTTCGGCTATCACTTTTGGATGGTCCC

GCGGCGTATTAGCTAGATGGTGGGGTAACGGCTCACCATGGCAATGATACGTAGCCGACCT

GAGAGGGTAATCGGCCACATTGGGACTGAGACACGGCCCAAACTCCTACGGGAGGCAGC

AGTAGGGAATCTTCCACAATGGACGAAAGTCTGATGGAGCAACGCCGCGTGAGTGAAGA

AGGGTTTCGGCTCGTAAAACTCTGTTGTTAAAGAAGAACATATCTGAGAGTAACTGTTCA

GGTATTGACGGTATTTAACCAGAAAGCCACGGCTAACTACGTGCCAGCAGCCGCGGTAAT

ACGTAGGTGGCAAGCGTTGTCCGGATTTATTGGGCGTAAAGCGAGCGCAGGCGGTTTTTT

AAGTCTGATGTGAAAGCCTTCGGCTCAACCGAAGAAGTGCATCGGAAACTGGGAAACTT

GAGTGCAGAAGAGGACAGTGGAACTCCATGTGTAGCGGTGAAATGCGTAGATATATGGAA

GAACACCAGTGGCGAAGGCGGCTGTCTGGTCTGTAACTGACGCTGAGGCTCGAAAGTAT

GGGTAGCAAACAGGATTAGATACCCTGGTAGTCCATACCGTAAACGATGAATGCTAAGTGT

TGGAGGGTTTCCGCCCTTCAGTGCTGCAGCTAACGCATTAAGCATTCCGCCTGGGGAGTA

CGGCCGCAAGGCTGAAACTCAAAGGAATTGACGGGGGCCCGCACAAGCGGTGGAGCATG

TGGTTTAATTCGAAGCTACGCGAAGAACCTTACCAGGTCTTGACATACTATGCAAATCTAA

GAGATTAGACGTTCCCTTCGGGGACATGGATACAGGTGGTGCATGGTTGTCGTCAGCTCG

TGTCGTGAGATGTTGGGTTAAGTCCCGCAACGAGCGCAACCCTTATTATCAGTTGCCAGCA

TTAAGTTGGGCACTCTGGTGAGACTGCCGGTGACAAACCGGAGGAAGGTGGGGATGACG

TCAAATCATCATGCCCCTTATGACCTGGGCTACACACGTGCTACAATGGATGGTACAACGA

GTTGCGAACTCGCGAGAGTAAGCTAATCTCTTAAAGCCATTCTCAGTTCGGATTGTAGGCT

GCAACTCGCCTACATGAAGTCGGAATCGCTAGTAATCGCGGATCAGCATGCCGCGGTGAAT

ACGTTCCCGGGCCTTGTACACACCGCCCGTCACACCATGAGAGTTTGTAACACCCAAAGT

CGGTGGGGTAACCTTTTAGGAACCAGCCGCCTAAGGTGGGACAGATGATTAGGGTGAAGT

CGTACAG

16S rDNA sequence of *Lactobacillus fermentum* ZJUIDS06 (1510bp), provided by Zhiyao Zheng:

CAGGATGAACGCCGGCGGTGTGCCTAATACATGCAAGTCGAACGCGTTGGCCCAATTGATTGATGGTGCTTGCACCTGATTGATTTTGGTCGCCAACGAGTGGCGGACGGGTGAGTAACACGTAGGTAACCTGCCCAGAAGCGGGGGACAACATTTGGAAACAGATGCTAATACCGCATAACAGCGTTGTTCGCATGAACAACGCTTAAAAGATGGCTTCTCGCTATCACTTCTGGATGGACCTGCGGTGCATTAGCTTGTTGGTGGGGTAACGGCCTACCAAGGCGATGATGCATAGCCGAGTTGAGAGACTGATCGGCCACAATGGGACTGAGACACGGCCCATACTCCTACGGGAGGCAGCAGTAGGGAATCTTCCACAATGGGCGCAAGCCTGATGGAGCAACACCGCGTGAGTGAAGAAGGGTTTCGGCTCGTAAAGCTCTGTTGTTAAAGAAGAACACGTATGAGAGTAACTGTTCATACGTTGACGGTATTTAACCAGAAAGTCACGGCTAACTACGTGCCAGCAGCCGCGGTAATACGTAGGTGGCAAGCGTTATCCGGATTTATTGGGCGTAAAGAGAGTGCAGGCGGTTTTCTAAGTCTGATGTGAAAGCCTTCGGCTTAACCGGAGAAGTGCATCGGAAACTGGATAACTTGAGTGCAGAAGAGGGTAGTGGAACTCCATGTGTAGCGGTGGAATGCGTAGATATATGGAAGAACACCAGTGGCGAAGGCGGCTACCTGGTCTGCAACTGACGCTGAGACTCGAAAGCATGGGTAGCGAACAGGATTAGATACCCTGGTAGTCCATGCCGTAAACGATGAGTGCTAGGTGTTGGAGGGTTTCCGCCCTTCAGTGCCGGAGCTAACGCATTAAGCACTCCGCCTGGGGAGTACGACCGCAAGGTTGAAACTCAAAGGAATTGACGGGGGCCCGCACAAGCGGTGGAGCATGTGGTTTAATTCGAAGCTACGCGAAGAACCTTACCAGGTCTTGACATCTTGCGCCAACCCTAGAGATAGGGCGTTTCCTTCGGGAACGCAATGACAGGTGGTGCATGGTCGTCGTCAGCTCGTGTCGTGAGATGTTGGGTTAAGTCCCGCAACGAGCGCAACCCTTGTTACTAGTTGCCAGCATTAAGTTGGGCACTCTAGTGAGACTGCCGGTGACAAACCGGAGGAAGGTGGGGACGACGTCAGATCATCATGCCCCTTATGACCTGGGCTACACACGTGCTACAATGGACGGTACAA

CGAGTCGCGAACTCGCGAGGGCAAGCAAATCTCTTAAAACCGTTCTCAGTTCGGACTGCAGGCTGCAACTCGCCTGCACGAAGTCGGAATCGCTAGTAATCGCGGATCAGCATGCCGCGGTGAATACGTTCCCGGGCCTTGTACACACCGCCCGTCACACCATGAGAGTTTGTAACACCCAAAGTCGGTGGGGTAACCTTTTAGGAGCCAGCCGCCTAAGGTGGGACAGATGATTAGGGTGAAGTCGTAC
