# Supplementary material for: Probiotic Effects of Lactobacillus fermentum ZJUIDS06 and Lactobacillus plantarum ZY08 on Hypercholesteremic Golden Hamsters
Source: Front Nutr. 2021 Jun 28;8:705763. doi: 10.3389/fnut.2021.705763 (PMC8273167; doi:10.3389/fnut.2021.705763)
Supplement: Supplementary file 2 [file Image_1.PDF]

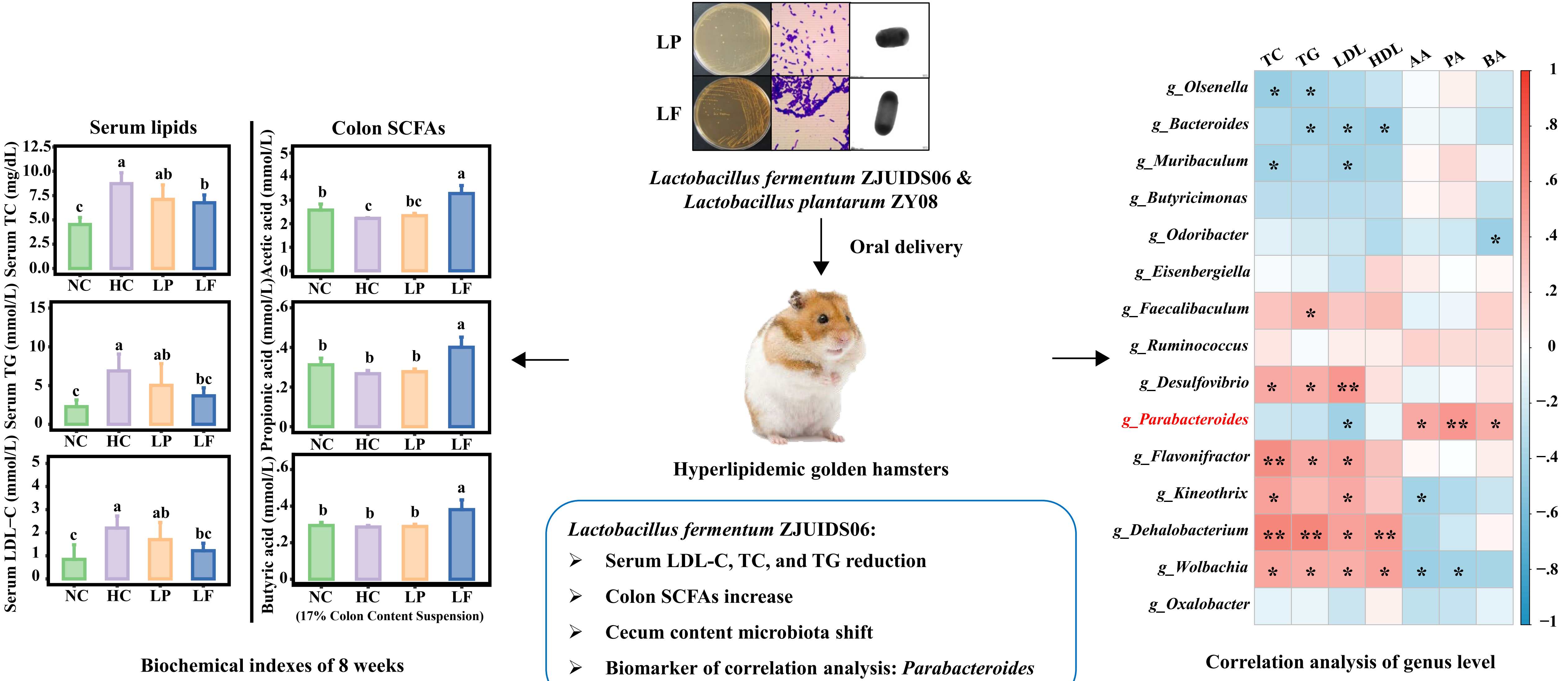
LP
LF
Lactobacillus fermentum ZJUIDS06 &
Lactobacillus plantarum ZY08
Oral delivery
Hyperlipidemic golden hamsters
Serum lipids
Serum TC (mg/dL)
Serum TG (mmol/L)
Serum LDL−C (mmol/L)
NC HC LP LF
Colon SCFAs
Acetic acid (mmol/L)
Propionic acid (mmol/L)
Butyric acid (mmol/L)
(17% Colon Content Suspension)
Biochemical indexes of 8 weeks
Lactobacillus fermentum ZJUIDS06:
Serum LDL-C, TC, and TG reduction
Colon SCFAs increase
Cecum content microbiota shift
Biomarker of correlation analysis: Parabacteroides
TC TG LDL HDL AA PA BA
g_Olsenella
g_Bacteroides
g_Muribaculum
g_Butyricimonas
g_Odoribacter
g_Eisenbergiella
g_Faecalibaculum
g_Ruminococcus
g_Desulfovibrio
g_Parabacteroides
g_Flavonifractor
g_Kineothrix
g_Dehalobacterium
g_Wolbachia
g_Oxalobacter
Correlation analysis of genus level
